# Pedunculated subserosal leiomyoma with torsion, a rare cause of abdominal pain: A case report

**DOI:** 10.1097/MD.0000000000032838

**Published:** 2023-02-03

**Authors:** Ching-Tang Chang, Sieh-Yang Lee, Ching-Di Chang

**Affiliations:** a Department of Emergency, Kaohsiung Municipal Ta-Tung Hospital, Kaohsiung City, Taiwan; b Department of Radiology, Kaohsiung Chang Gung Memorial Hospital, Chang Gung University College of Medicine, Kaohsiung City, Taiwan.

**Keywords:** abdominal pain, Leiomyoma, torsion

## Abstract

Acute abdominal pain caused by torsion of the pedunculated subserosal leiomyoma is rare. Miss diagnosis can lead to ischemia, necrosis, and subsequent peritonitis which may cause significant morbidity. Torsion of the leiomyoma should be recognized as a surgical emergency and once suspected, early surgical intervention should be considered.

**Patient concerns:**

A 28-year-old woman with a medical history of uterine leiomyoma presented to our emergency department because of acute onset right lower abdominal pain.

**Diagnoses:**

The computed tomography was performed which demonstrated multiple leiomyomas of variable sizes and a subserosal leiomyoma located at right lower abdomen with poor contrast enhancement.

**Interventions:**

The gynecologist was consulted, and myomectomy was performed. The intraoperative finding showed a pedunculated subserosal leiomyoma with torsion.

**Outcomes:**

She underwent myomectomy for the twisted pedunculated subserosal leiomyoma as well as other leiomyomas and was discharged with a favorable outcome.

**Conclusions:**

Torsion of the leiomyoma is a surgical emergency as delayed in treatment may lead to marked morbidity. Once suspected, the gynecologist must be consulted, and surgical intervention should be considered.

## 1. Introduction

Uterine leiomyomas, also known as uterine fibroids, are by far the most common pelvic tumor in females of reproductive age.^[1]^ Wide range in prevalence was reported (4.5%–68.6%) based on the study populations and the imaging modalities.^[2]^ It is a benign tumor arising from the smooth muscle cells of the myometrium. Intrauterine leiomyomas are generally classified as submucosal, intramural, and subserosal according to their location in the uterus.^[3]^ The symptoms of uterine leimyomas are often chronic and may range from asymptomatic to abnormal uterine bleeding, bulk-related symptoms, and reproductive dysfunction.^[4]^ Acute abdominal pain caused by torsion of the pedunculated subserosal leiomyoma is rare and is often difficult to diagnose preoperatively. Miss diagnosis can lead to ischemia, necrosis, and subsequent peritonitis which may cause significant morbidity.^[5,6]^ As a result, torsion of a pedunculated subserosal leiomyoma should be recognized as a surgical emergency. Once suspected, the gynecologist must be consulted, and early surgical intervention should be considered.

## 2. Case report

**Figure 1.**
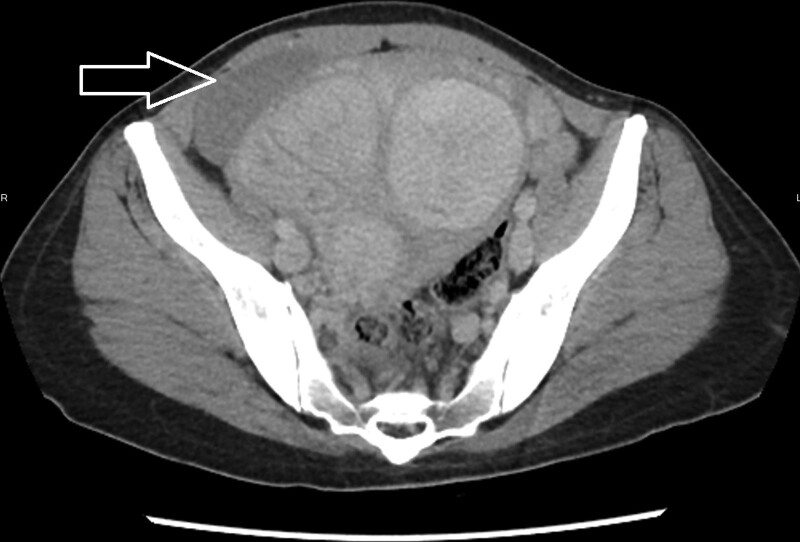
Axial view of CT imaging with contrast enhancement showed a poor contrast enhancement subserosal leiomyoma on right uterine surface (arrow). CT = computed tomography.

**Figure 2.**
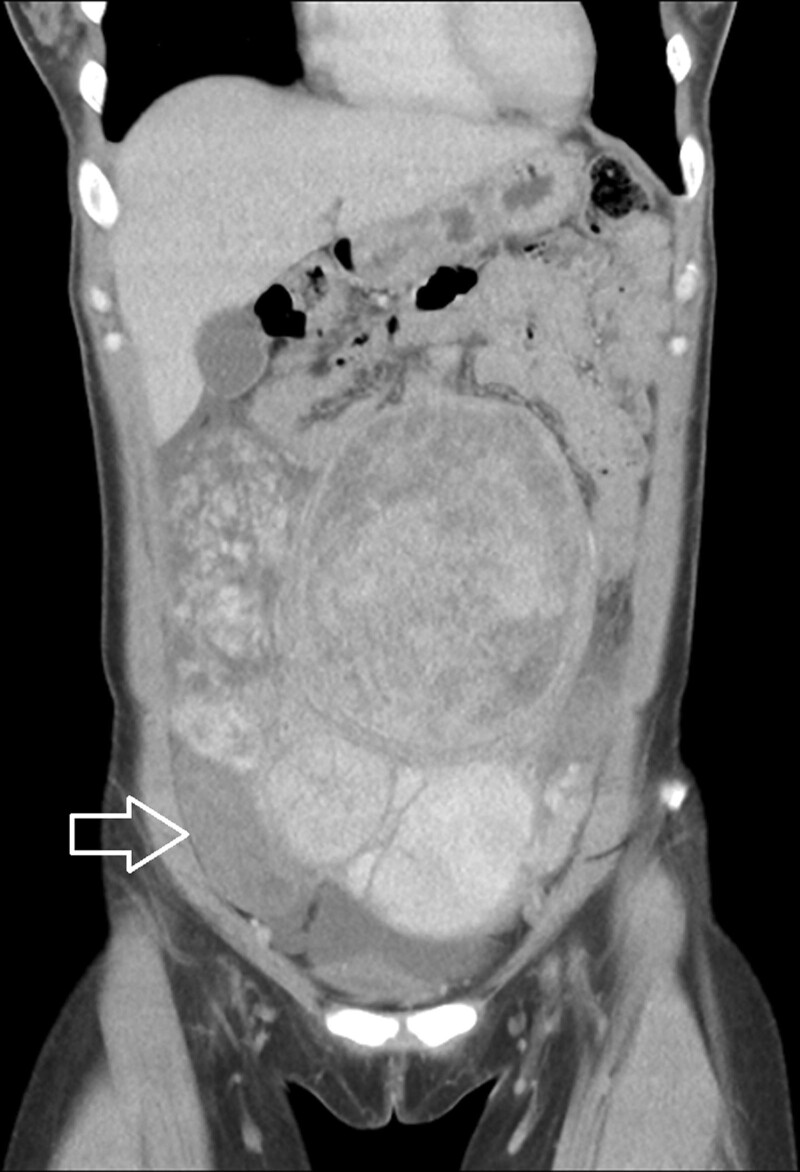
Coronal view of CT imaging with contrast enhancement showed a poor enhancement subserosal leiomyoma (arrow). CT = computed tomography.

**Figure 3.**
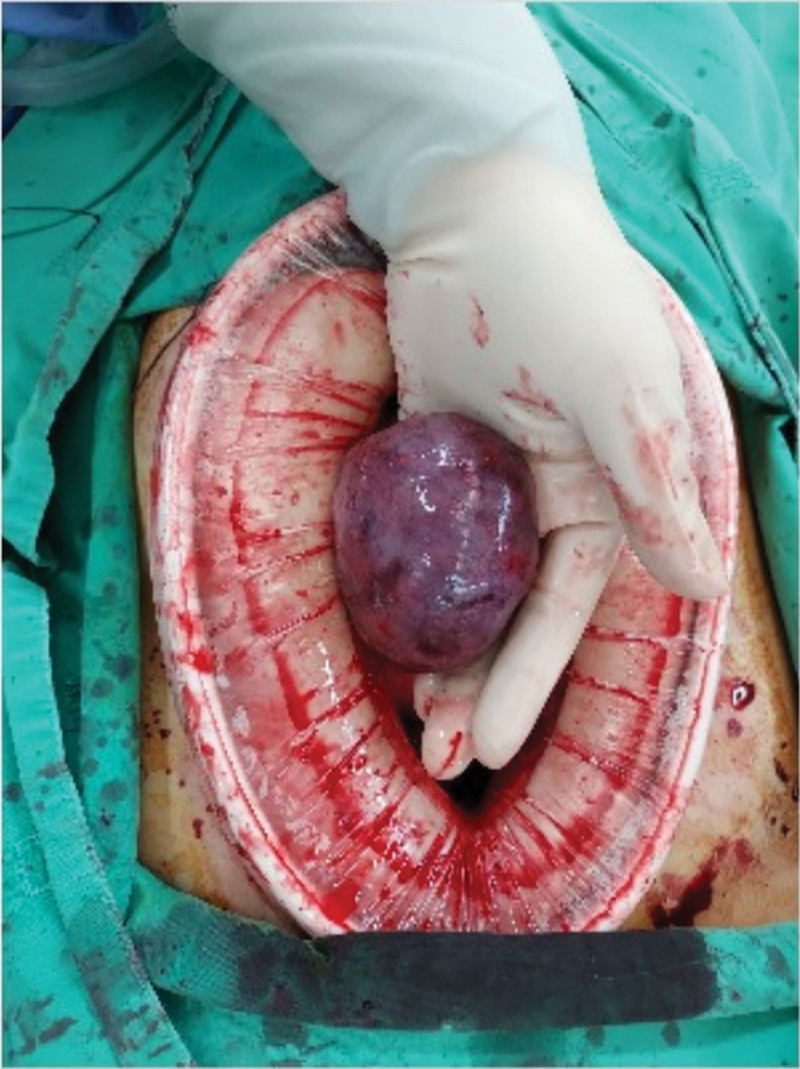
The intraoperative finding showed a pedunculated subserosal leiomyoma with torsion and ischemic change.

A 28-year-old woman with a medical history of uterine leiomyoma presented to our emergency department because of acute onset right lower abdominal pain. The vital signs were stable without fever and there were no gastrointestinal tract or urinary symptoms. On physical examination, there was marked right lower abdominal tenderness. Laboratory data revealed WBC 10830, Hb 12.6 g/dL, C reactive protein 0.75 mg/L, and negative pregnancy test. Urine analysis showed only mild hematuria. Due to intractable pain, abdominal computed tomography (CT) was performed which demonstrated multiple leiomyomas of variable sizes and a subserosal leiomyoma located at right lower abdomen with poor contrast enhancement (Figs. 1 and 2). There were no imaging features of appendicitis, diverticulitis, bowel obstruction, or high attenuated ascites. The gynecologist was consulted, and myomectomy was performed. The intraoperative finding showed a pedunculated subserosal leiomyoma with torsion (Fig. 3). She was discharged uneventfully after the surgery without complications.

## 3. Discussion

Uterine leiomyomas are the most common pelvic tumors in female of childbearing age. The clinical symptoms are usually chronic, and may range from asymptomatic to abnormal uterine bleeding, dysmenorrhea, menorrhagia, pelvic pain, frequent urination, and reproductive dysfunction. Acute abdominal pain caused by leiomyomas rarely occurred and alternative diseases originate from nearby organs should be considered and evaluated carefully.

It may be challenging to diagnose a pedunculated subserosal leiomyoma with torsion preoperatively. Ultrasound is the most widely used imaging modality for evaluating leiomyomas due to its availability and cost-effectiveness. Torsion of the pedunculated subserosal leiomyoma should be suspected if color Doppler demonstrated a twisted pedicle or decreased vascular supply. However, ultrasound may be a less effective method if the twisted pedicle is thin or invisible.^[7]^ The CT with contrast enhancement may be another alternative modality especially in the emergency settings for the evaluation of abdominal pain. The typical CT findings of a pedunculated subseroal leiomyoma with torsion may include poor contrast enhancement and congestion of the vascular pedicle at the site of torsion.^[8]^ In addition, the CT images are also useful to exclude other possible causes of abdominal pain. As a result, the role of CT scan in this clinical setting may be regarded as the first-line diagnostic tool for making the correct diagnosis and to exclude other possible life-threatening disease. Magnetic resonance imaging (MRI) is the most effective modality for visualizing the size and location of the uterine leiomyomas. Identifying a necrotic leiomyoma on the MRI and specifically with the presence of a pedicle between the leiomyoma and uterus is suggestive of torsion.^[8]^ However, because MRI requires a more stable patient, it is time-consuming to perform the examination, and relative lack of availability, the MRI may be difficult to apply in the emergency department for the evaluation of abdominal pain in regular daily practice.

In our patient, the contrast enhanced CT scan is performed for the evaluation of acute and intractable abdominal pain. Serious lesions were excluded except a hypo-perfused subserosal leiomyoma. Based on the clinical presentation, laboratory study, and the image finding, ischemic pain from the subserosal leiomyoma was highly suspected. She underwent myomectomy for the twisted pedunculated subserosal leiomyoma as well as other leiomyomas and was discharged with a favorable outcome.

## 4. Conclusion

Although rare, torsion of the pedunculated subserosal leiomyoma must be considered in females presenting with an acute onset lower abdominal pain. The diagnosis should base on the clinical presentations and image findings. Torsion of the leiomyoma is a surgical emergency as delayed in treatment may lead to marked morbidity. Once suspected, the gynecologist must be consulted, and surgical intervention should be considered.

## Author contributions

**Writing – original draft:** Ching-Tang Chang.

**Writing – review & editing:** Ching-Di Chang, Sieh-Yang Lee.
